# Nursing Process implementation in a gerontogeriatric context: qualitative research

**DOI:** 10.1590/0034-7167-2023-0465

**Published:** 2024-09-06

**Authors:** Francine Casarin, Juliana Silveira Colomé, Liliane Alves Pereira, Emanuelli Mancio Ferreira da Luz, Oclaris Lopes Munhoz, Silomar Ilha

**Affiliations:** I Faculdade Integrada de Santa Maria. Santa Maria, Rio Grande do Sul, Brazil; II Universidade Franciscana. Santa Maria, Rio Grande do Sul, Brazil; III Universidade Federal do Rio Grande. Rio Grande, Rio Grande do Sul, Brazil; IV Universidade Federal de Santa Maria. Palmeira das Missões, Rio Grande do Sul, Brazil

**Keywords:** Aged, Geriatrics, Nursing Care, Nursing Process, Nursing, Anciano, Geriatría, Atención de Enfermería, Proceso de Enfermería, Enfermería

## Abstract

**Objective:**

to describe Nursing Process implementation in a faith-based senior living community.

**Method:**

strategic action research with 19 nursing professionals and three managers of a faith-based senior living community. Implementation took place in four phases: diagnosis, planning, implementation and assessment. The data collected through semi-structured interviews and focus groups were subjected to discursive textual analysis.

**Results:**

the central categories were constructed: Nursing Process in faith-based senior living community: diagnosis of knowledge and application; Nursing Process in faith-based senior living community: implementation; Nursing process in faith-based senior living community: assessment after its implementation.

**Conclusion:**

Nursing Process implementation made it possible to structure work management/organization, contributing to knowledge, organization and continuity of care for safety and professional support.

## RESUMEN

**Objetivo:**

describir la implementación del Proceso de Enfermería en una residencia religiosa para mujeres adultas mayores.

**Método:**

investigación de acción estratégica con 19 profesionales de enfermería y tres gestores de una residencia religiosa para ancianas. La implementación se desarrolló en cuatro fases: diagnóstico, planificación, implementación y evaluación. Los datos recopilados a través de entrevistas semiestructuradas y grupos focales fueron sometidos a análisis textual discursivo.

**Resultados:**

se construyeron las categorías centrales: Proceso de Enfermería en la residencia religiosa: diagnóstico de conocimientos y aplicación; Proceso de enfermería en la residencia religiosa: implementación; Proceso de enfermería en la residencia religiosa: evaluación tras su implementación.

**Conclusión:**

la implementación del Proceso de Enfermería permitió estructurar la gestión/organización del trabajo, contribuyendo al conocimiento, organización y continuidad de los cuidados para la seguridad y el apoyo profesional.

## INTRODUCTION

The discussion about population aging has occurred worldwide in recent years, reflecting the significant increase in the number of older adults. Projections indicate that, by 2050, there will be two people over 65 for every one up to four years old in the world^(1)^. I n the Brazilian reality, this debate is emerging, given that, in 2025, Brazil will be the sixth largest country in terms of concentration of this population^(2)^. As aging occurs, individuals become susceptible to social and/or family vulnerabilities that can predispose to geriatric syndromes, such as cognitive/communicative disability, postural instability, sphincter incontinence, immobility and family insufficiency, resulting in the need for healthcare^(2)^.

From this perspective, care for older adults can be carried out in different modalities, such as in hospital settings, in Nursing Homes (NHs) and in homes. The home environment is considered, in most cases, the place where older adults spend most of their time. It is common for them to feel comfortable and safe there. Therefore, it must be a space that provides not only well-being, but the fundamental conditions in the daily care of older adults with family members and/or caregivers^(4)^.

Care for religious older adults is structured in faith-based senior living communities, which are environments where sisters who need assistance with their senescence (physiological process of aging) or senility (associated with a disease condition) live. They are chosen because they provide tranquility, comfort and welcome for older adult sisters or those who have limitations that make it impossible for them to reside in other communities^(5)^.

These institutions must meet this population’ needs, consider their life trajectory and preserve their autonomy and independence, promoting understanding of the aging process and making them major players of their care^(5)^. Thus, the need for professionals to act with a view to interprofessional care emerges. Among these, the nursing team provides direct and comprehensive care for older adults, under nurses’ management, who need to carry out their work activities in increasingly organized and structured environments^(6)^.

In this context, the Nursing Process (NP) emerges, a method guiding nurses’ critical thinking and clinical judgment, with a view to directing the nursing team towards care, through five stages: nursing assessment, diagnosis, planning, implementation and evolution^(7)^. In Brazil, NP has been the subject of extensive discussion regarding nurse performance. Although there is a legal framework regarding its obligation in every socio-environmental context in which nursing care occurs^(7)^, there is still a limited understanding of the concept and its use and application. In this regard, it is possible to identify that, in some realities, NP is implemented more out of obligation than by understanding its need and relevance for nursing care quality. This fact makes it difficult to understand the practical implications of its use.

To this end, there is research on the subject in different countries, such as Ethiopia^(8)^, Nigeria^(9)^, China^(10)^, Japan^(11)^ and Brazil^(12)^. However, there is a lack of research on the steps for its effective implementation in home care settings aimed at religious older adults. Given this, this research is necessary because it presents the itinerary followed in its implementation, aiming at the quality of humanized and unique care, conceiving the biopsychosocial and spiritual processes of older adults. Given the above, the question arises: how to implement Nursing Process in a faith-based senior living community?

## OBJECTIVE

To describe Nursing Process implementation in a faith-based senior living community.

## METHODS

### Ethical aspects

This research was authorized by the institution and approved by the *Universidade Franciscana* Research Ethics Committee. Participation in the research occurred by signing the Informed Consent Form (ICF). Participants were assured of information anonymity and confidentiality and identified by the letter P (Participant), followed by a number.

### Study design

This is strategic action research, in which the transformation is previously planned by the researcher, who monitors the effects and assesses the results of its application^(13, 14)^. To this end, the phases of diagnosis, planning, implementation and assessment were followed, which include the eight steps of action research: problem identification; data collection; data analysis; data meaning; identification of needs for change; finding solutions; intervention action; and transformation^(14)^. Regarding compliance with methodological rigor criteria for writing the research, the Consolidated criteria for REporting Qualitative research (COREQ) was used^(15)^.

### Study setting and period

This research was carried out between February and October 2022 in a faith-based senior living community in the central region of the state of Rio Grande do Sul, Brazil, founded on March 25, 1951, which offers care to nuns at the *Instituto das Irmãs Franciscanas da Penitência e Caridade Cristã,* which has two wings, where 105 older adults with different levels of dependence reside. The place maintains itself with its own resources, medications via the State and retirement benefit for older adults.

### Population

During the period investigated, 39 workers in care and non-care services worked in the setting, including four nurses, 17 nursing technicians, an assistant doctor, a volunteer doctor, a nutritionist, one physiotherapist, two hygiene professionals, nine pantry workers and three management professionals. Nursing and management professionals working during the data collection period and who met the selection criteria were considered.

### Participant selection criteria

Nurses, nursing technicians and/or management professionals who have worked in a faith-based senior living community for at least one month, a period sufficient for them to have already experienced the reality of the institution, were included. Professionals who were on leave for any reason during data collection were excluded. The choice of the nursing team comes from the fact that they are the professionals responsible for operationalizing the NP^(7)^. Management professionals were included due to the understanding that, for the successful implementation of a new work methodology, it is necessary for them to fully understand it and recognize it as necessary. Thus, of the 24 eligible participants, 22 met the criteria.

### Data collect

Data were collected using semi-structured interview and focus group (FG) techniques in four phases: diagnosis (February to April 2022); planning (August 2022); implementation (September 2022); and assessment (October 2022). All stages were developed in a reserved location at the research institution itself.

#### Diagnosis

In the diagnostic phase, initial data was collected through a semi-structured interview (1^st^ moment), with 19 nursing professionals and three management professionals. The interview script was composed of participant description and open-ended questions: how is the care process for older adults developed in the place where you operate? What do you understand by NP? Have you already experienced/developed NP with older adults living in the faith-based senior living community where you work? Interviews were carried out individually and with prior appointment by two trained professionals (a nurse with experience in qualitative research, NP and gerontology; and a scientific initiation student who received training). The interviews lasted an average of 60 minutes.

The interview enabled researchers to identify the lack of NP in the investigated setting, absence of manuals, protocols, Standard Operating Procedures (SOPs) and weaknesses in knowledge about NP, which led to the need for awareness-raising workshops on the topic in conceptual aspects and application. Four workshops were held, from March to August 2022, with the 22 professionals participating in the research, led by a research nurse and specialist in gerontology. They included a dialogued expository presentation and a conversation circle, moments in which participants were encouraged to ask questions, resolve doubts and handle material linked to NP^(16)^. Subsequently, new interviews were carried out with participants (2^nd^ moment), from May to August 2022, with the same script as the previous interview, to assess workshops’ contributions in building the knowledge of nursing and management professionals about the object of study.

#### Planning

The planning phase occurred after awareness raising, through two FGs with 19 nursing professionals, with a view to building the instrument and guiding materials for NP development. Only nursing professionals were considered for this moment, as they are responsible for developing the NP.

In the first FG, the 14 participants received a copy of COFEN Resolution 358/2009 (valid at that time, replaced in 2024 by COFEN Resolution 736/2024)^(7)^ and were invited to read it collectively. Afterwards, they discussed how to systematize an NP facilitating instrument. The FG was finalized with a synthesis of suggestions for the NP instrument construction. Moreover, the need to create manuals with information regarding the main care, daily routines, standardization of abbreviations for nursing records and SOPs emerged. These signs were compiled to construct the materials and prototype of the NP instrument in the setting.

For the second FG, there were 15 participants, to whom the moderator gave a copy of the NP’s guiding instrument. After approval by professionals, the group discussed its implementation. It was approved that each older adult would have an identified clipboard, which would remain at the nursing station, containing a medical prescription, NP instrument and nursing record. The times for operationalizing the NP stages were decided. Subsequently, training was carried out with NP instrument simulation and standardization of nursing checks and records. It was advised that using the NP prototype would begin on 09/05/22 at 00:00, with use by the team for 30 days. After this period, it would be assessed in a new FG. Finally, the meeting was summarized based on the topics discussed, and professionals were guided on the implementation of the prototype built to guide the NP.

#### Implementation

This stage occurred through the use, by nursing professionals, of the NP guiding instrument in daily care in the investigated setting. A FG was also held with the three nurses to assess and contribute to the materials created to assist the NP, work process organization and NANDA-I study^(16)^. At first, the moderator presented a physical copy of each of the materials created by the researchers (based on the materials compiled from the second FG), which were named: Manual of Main Care/Procedures; Nursing Care Daily Routines Manual; Abbreviations and Standardized Acronyms for Nursing Records; and SOPs.

In the second moment, the NANDA-I^(16)^ was discussed in physical format to delve deeper into the domains, classes and diagnoses. Afterwards, nurses selected the main nursing diagnoses that represented the study population’s health conditions. These were transferred to a Microsoft Word® chart, which remained available on the computer for nurses to consult. Finally, this document was validated in collective synthesis.

At the end of this phase, one of the researchers, together with the professional responsible for the institution’s Information Technology, computerized the NP instrument and adapted the institution’s system to take into account nursing evolution, diagnoses and prescriptions. Also, one of the researchers met with the local Regional Nursing Council (COREN - *Conselho Regional de Enfermagem*) inspector to present the NP proposal, as, in the setting studied, it was planned on a weekly basis, requiring legal approval. The COREN inspector approved the proposal, considering that the NP’s premise is adaptability to meet the needs of each institutional reality.

#### Assessment

Finally, in the assessment phase, the fourth FG took place with the 19 nursing professionals, at which time the materials were presented, such as basic macroprocesses and microprocesses related to NP. It was suggested that participants reflect on “How can these instruments help NP in the daily life of a faith-based senior living community?”. The reflection was recorded on A4 sheets of paper and with pens. In a second moment, participants were encouraged to reflect on using the NP prototype, which had already been implemented for 30 days in a faith-based senior living community. Thus, on the other side of the sheet distributed previously, they recorded the strengths and weaknesses they experienced when using it, which was presented to the other participants.

All FGs took place in the faith-based senior living community’s main auditorium, a large and comfortable place, free from the influence of external noise, with chairs arranged in a circle, to encourage interaction. They were conducted by one of the researchers, as a mediator, with the role of organizing and coordinating the FG, and a scientific initiation student, as an observer, who assisted in the process of recording the speeches, notes and the dynamics carried out.

### Data processing and analysis

The researched statements, obtained in interviews and FGs, were audio recorded with an electronic MP3 audio device and transcribed in full mechanically by the researchers in Microsoft Word®. The technique of discursive textual analysis was used, organized in unitarization, establishment of relationships and communication^(17)^. Initially, the researchers examined the texts with intensity and depth, forming three central categories, based on knowledge diagnosis and NP application, planning, implementation and assessment. Afterwards, each report was read and separated into different units of meaning and, finally, separated into ten categories. Finally, the understandings achieved from the two previous focuses through the communication process were presented, resulting in metatexts describing and interpreting the phenomena investigated^(17)^.

### RESULTS

Of the 22 participants, all were female, three nurses, 16 nursing technicians and three management professionals, aged between 19 and 62 years. Training time ranged from 45 days to 36 years, and professional experience ranged from a minimum of 30 days to a maximum of 30 years. The time spent working at a faith-based senior living community ranged from 30 days to seven years.

The data produced in the interviews comprised the diagnostic phase and resulted in a central category and three categories of analysis. The first three FGs comprised the planning and implementation phases, resulting in a central category and three analysis categories. The fourth FG comprised the assessment phase of the NP implemented in the setting, resulting in a central category and four analysis categories, as shown in Chart 1.

**Chart 1:** Summary of data analysis steps. Santa Maria, Rio Grande do Sul, Brazil, 2022

Nursing Process in faith-based senior living community	
Central category	Analysis category
Knowledge diagnosis and application	- Presence of a routine, absence of the Nursing Process. - (Lack of) Knowledge about the Nursing Process before and after awareness workshops. - Understanding the importance of the Nursing Process.
Planning and implementation	- Perspectives for Nursing Process implementation. - Feeling part of the process. - Organization of necessary instruments
Assessment after implementation	- Quality of care: knowledge, organization and continuity. - Quality of care: safety and professional support. - Time in recording records: weakness to be overcome. - Care beyond the biological: need for time for contact and coexistence with older adults.

### Presence of a routine, absence of the Nursing Process

During the diagnostic phase of the research, it was clear that participants identified the presence of a care routine for older adults, but were unaware of the existence of NP.

*Care was carried out, in the vast majority of cases, and we, technicians, who organized it as a group and divided the tasks. We only write the evolutions in the notebook if something in sisters’ routine changes.* (P1)

*There is nothing manual, what we do is as the person wants. Now that we have the nurse, we do as she asks us to do. There is nothing to guide our development, there is nothing to tell us how to do it.* (P3)

*It’s always that routine. We follow the guidance of the nurse and the coordinator, who are always there together. There’s no manual, SOP, I’ve never seen it, at least. Guidance is given during shift changes or when we need something.* (P5)

*I think there is a routine that we always try to follow, always prioritizing the sisters’ well-being, taking care of them.* (P21)

### (Lack of)Knowledge about the Nursing Process before and after awareness workshops

Still as a diagnosis, before the awareness workshops, it was possible to identify that participants were unaware of the NP concept and applicability.

*I remember it’s about patient care: taking care of others, dedicating yourself, that’s what I remember from when I was studying.* (P1)

*Look, I’ve never heard of it, but the name says it, it must be a management system, more or less that. I understand the word, because I’ve never heard of it. It would be a management, conduct and action management system, management of everything.* (P18)

*I don’t understand, I haven’t heard of it, it’s a system for all nursing.* (P19)

After raising awareness, participants had a better understanding of the NP, as the responses were more coherent with the literature and resolutions, as follows:

*The Nursing Process involves the technician. The Nursing Process, from what I understand, is organized by the nurse: they visit patients, see what they have to do for patients to improve. Thus, they organize care into five stages, which are data collection, diagnosis, planning, implementation and assessment. We, technicians, carry out the care prescribed by the nurse.* (P1)

*The Nursing Process is assistance with the sisters. It starts with data collection, nursing diagnosis, then planning, setting an objective based on patient assessment and, on top of that, we will carry out the actions. Based on what the nurse assesses this process, they will plan, prescribe and, in the intervention, the techniques will be performed, and then the nurses will reassess again.* (P18)

*The Nursing Process is carried out by the nurse and we (technicians) develop the care. The nurse takes the history and physical examination. The nurse who prescribes the care, and we, technicians, will do the care part. The Nursing Process is care. The nurse goes to the patient and performs anamnesis, talks, and takes notes, then a physical examination, they observe, then check NANDA diagnoses, prescribe care, and the technician goes to the development part. Then an assessment is made to see if it is progressing well. The nurses prescribe and we carry out most of the care.* (P19)

### Understandings about the importance of the Nursing Process

From awareness raising, participants’ better knowledge and understanding of the importance of the NP in their daily work with older adults was perceived.

*It is extremely important. Care records are as important as the care provided, as there are still some improvements to be made.* (P2)

*I understand the importance of the house as an instrument of guidance in the professional practice of each professional in the house as a guide who is helping us to provide quality work for each patient/sister in the house. It is important to be able to visualize what each patient says and the method in which the work will be organized.* (P5)

*I realize that the Nursing Process is very important. Nursing records are the guarantee of our service that we perform, organization in the house and of each one to provide good care to the sisters.* (P6)

*The Nursing Process, established in the home, is extremely important so that there can be continuity and legitimacy of the care provided. The process guides the nursing team in carrying out activities inherent to the profession, allowing us to improve and develop techniques and skills already acquired, share doubts and suggestions, aiming at the health and care of each older adult.* (P11)

### Perspectives for Nursing Process implementation

In the planning and implementation phases, the perspectives raised by the team regarding NP implementation in a faith-based senior living community were identified. The understanding that implementation would assist in continuity of care, would bring more safety, organization and better care to older adults, as well as greater visibility, trust and team fulfillment stood out.

*After implementing the Nursing Process, there will be continuity of care already provided and organization, even greater care, greater attention.* (P7)

*It will bring more safety, organization, a method for the care provided, visibility into more organized nursing records, specific to each patient, for more accurate and safe nursing care. Carrying out the care that we, technicians, will carry out, but according to nurse prescription and nursing consultation, will bring more safety and quality to care, greater organization also for the environment.* (P9)

*For a satisfactory result, we need the correct process and actions; this is what this process is providing us, helping us to have more confidence and fulfillment.* (P12)

*It will qualify care, through the implementation of the process, with the use of reports in routines. Each work carried out will be accompanied by a date, time, who carried it out, and a stamp by the responsible technician, giving us support for the work carried out. Teamwork with knowledge exchange.* (P14)

### Feeling part of the process

Participants were happy to be part of NP planning and implementation in a faith-based senior living community. They highlighted the learning for the team, the improvement in the daily care routine and greater visualization of the assistance provided, according to the following reports.

*I feel privileged to be part of this process, to have the opportunity to acquire knowledge and increasingly improve my functions, my learning, to develop serious, responsible and humanized work.* (P2)

*Being part of this process is a great challenge, both for professional development and for skill and care development, it is to be a comprehensive and fundamental part of the Nursing Process as a whole.* (P11)

*I feel safe having support from the Nursing Process. I am adapting, I feel happy to be part of this experience, which is certainly enriching for us on the nursing team and, for me, as a professional in this area.* (P13)

*Constant learning, perhaps a greater commitment to daily activities, responsibility, agility and satisfaction in learning more and more.* (P15)

### Organization of necessary instruments

During the planning and implementation phases, materials were created and approved to assist the NP. As shown below, professionals stated that such materials can clarify doubts about care, standardize and professionalize work, which will result in greater safety and better care for older adults.

*The materials created to assist are great and will help with care to answer questions on a daily basis.* (P5)

*The materials are fundamental and it’s a difference, because we didn’t have anything like that here before. They are very important, because we can consult and answer questions about care and standard acronyms to use in records.* (P6)

*I think that having separate clipboards with each sister’s name, containing the Nursing Process record and instrument, will be very good, as it will professionalize our work, further improving sister care.* (P16)

*They are very important and were very illustrative and in easy-to-understand language. SOPs, then, of course, have standardized a lot of care and this brings more safety for us and for the sisters too.* (P17)

### Quality of care: knowledge, organization and continuity

After 30 days of NP implementation, contributions to quality of work, greater knowledge, work process organization and absence of fragmentation of care were noted.

*This methodology helped to get to know the sisters better. it’s easier to see what you have to do. With the prescription, you can see if you continue with the care or if you need another one, so it is easier for us to observe/visualize and carry out care. And the team’s relationship makes communication easier, we can work together very well. if I forget something, the team helps and this improves quality for the sisters.* (P8)

*Clinical nursing care with each sister. Our work as a team has changed. Today, we are able to have continuity of work, communication, sister care, a clinical look at each one, every change, fragility that happens between them, that’s it.* (P14)

*The nurse’s work has greater visibility and all care is applied and checked. Sometimes, I stop looking and the quality of the evolutions is much better. They check and evolve; the quality of the techniques’ work is much better.* (P20)

*More organized care, greater visibility; having individual clipboards with nursing prescriptions makes it easier to see the care to be carried out with the sisters. Then, we are able to visualize what was done in the previous shift.* (P16)

### Quality of care: safety and professional support

Professional support and safety were also potentialities reported by participants during the assessment phase of NP implementation, as follows:

*I think it helped a lot when it came to evolution and procedures. There is greater security in the procedures carried out by the previous team.* (P17)

*Greater support, because it helps when we forget to take care of ourselves. We look and help you to know what to do.* (P7)

*The nursing prescription brings us, in addition to professional support, greater knowledge, opportunities for communication with nurses and also guidance regarding the care for our patients.* (P2)

*Safety in caring for sisters, care provided is care checked. Be supported there through records. And with prescriptions, we can read and know what care we need to take, what product to use for dressings, for instance. Better than just passing the shift, we have that support.* (P22)

### Record execution time: weakness to be overcome

One of the weaknesses to be overcome during NP implementation in a faith-based senior living community, evidenced in the assessment process, concerns the time to execute nursing records.

*Time for us is very complicated. The first ones I did took me three hours. Sometimes, when you’re doing a physical exam and something goes wrong with a sister, you have to drop what you’re doing and see your sister.* (P20)

*For me, it only has benefits, really. But there is a weakness, which I noticed, which is the time to record care. One day it took me three hours and 20 minutes, but now I’m slowly improving.* (P2)

*Lack of time to fill out documents; question of time itself* (P7).

*There is no time to complete the nursing record. Sometimes, we are late for the shift change, because one calls, another calls, so it’s time.* (P5)

### Care beyond the biological: need for time for contact and coexistence with older adults

During the NP assessment process, professionals reported that, due to the time required to complete nursing records, they were not able to stay with older adults for a long time, which represented a fragility.

*Lack of time to pay more attention to the sisters to provide quality care.* (P6)

*The issue of time, because we, technicians, don’t have much time to interact with the sisters, because we have to evolve everything that has been done with care.* (P10)

*The issue of time with the sisters is very busy when we are alone. Sometimes the sisters call us, they want to talk, but we have to evolve, check. I think it’s more of a tension, because, between one care and another, we talk, but it’s all in a rush.* (P19)

*Pay attention to the sisters, time to talk to them, have more dialogue. Sometimes it is more difficult, due to the demands of daily tasks. Pay attention to the sisters, the moment of dialogue, of talking, this has left a little to be desired.* (P22)

## DISCUSSION

The interface between the NP and nurses’ work process is effective based on the understanding of this as a guiding method for critical thinking and nurses’ clinical judgment^(7)^. As nurses take ownership of the NP, it is possible to assess the level of organization, highlight possibilities for improvements and carry out situational diagnosis, the basis of the nursing service strategic planning, making it possible to assess the level of organization of the care provided^(12)^.

Although more than 14 years have passed since the publication of a resolution that presents the obligation of NP, its conceptual clarification and practical operationalization remain challenging in some nursing practice settings^(12)^, evidenced by nursing care settings that have not yet implemented it. Especially in the context of faith-based senior living communities, it is clear that the resolution™, in itself, may not have offered the support that NP operationalization requires, as many factors triggered practical difficulties in implementing and implementing the NP steps, which was modified with this action research.

In the context of this research, nursing professionals had an empirical work routine regarding how to carry it out, the human resources involved and work instruments. Similar data was evidenced in a study with religious older adults in a NH in Rio Grande do Sul^(18)^, where care was directed to older adults’ needs; however, it was conducted based on care standards and routines, without using a specific and solid method^(18)^. Among the factors that contribute to fragmentation of care in places that do not have the NP established, we can mention work overload, the difficulty of working as a team, insufficient qualifications and absence of materials^(19)^. In the home care environment, nurses have skills and competencies to provide, together with the multidisciplinary team, quality assistance, making older adults the leading actors of their care^(20)^.

In this regard, during the interviews with the participants of this research, it was evident that they were unaware of the concept and applicability of NP in the daily care practice of older adults. This diagnosis signaled a warning that it was not being developed at the institution. Thus, a study with nursing professionals working with older adults identified that participants recognized the method of guiding care for this population. However, they had difficulties in conceptualizing it, given the lack of knowledge about the importance of NP in daily work, resulting in a fragmented practice based on routines^(19)^. To this end, professional qualification, ongoing education and continuing education of the team in using NP are essential. With this, it will be possible to expand professionals’ knowledge on the subject so that care is not limited to everyday practices and demands^(21)^.

It was identified that awareness-raising workshops contributed to participants’ understanding of the NP and the importance of its use in daily work. Investing in knowledge-driving methodologies is necessary to help modify attitudes and work processes. From the knowledge and understanding of its importance, it will be possible to develop the NP stages more effectively. From this perspective, interactive learning experiences are important and favor clinical nursing practice^(10)^.

The correct and effective NP application scientifically supports nursing care and brings professionals closer to people who need assistance. For nursing performance to improve, those involved need to be aware and understand that nursing care management is part of comprehensive management. Furthermore, actions must be planned and executed at all phases of care. Therefore, the NP is essential for practice in terms of guaranteeing autonomy and strengthening the professional category^(8)^.

Regarding the interface between theory and practice in academic training, the conceptual understanding of NP deserves to be highlighted and should be encouraged as it favors adequate and quality care and is a mandatory method that favors a unique perspective on patients. Having knowledge on the subject contributes to recognizing nursing as a scientific profession with a social character^(8)^.

The construction of instruments to strengthen NP implementation proved to be positive and promising in the assessed context, as it contributed to greater support and safety for professionals, work process organization and absence of fragmentation of care in daily life. In addition to raising awareness, there were improvements in the perception of NP and changes in professional attitudes and the work process, which enhanced the consolidation of the constructed instruments and the NP in a faith-based senior living community. Therefore, it can be inferred that repercussions on quality of care will also occur, since the NP improves care and offers legal support for the profession through its records, in addition to granting autonomy to professionals, promoting the visibility of the profession.

Through the NP, nurses’ role and autonomy are consolidated in the application of their knowledge and in achieving recognition for the quality of care provided. Therefore, nursing professionals, when using the NP, feel prepared and safe to carry out their activities, in addition to perceiving themselves with greater professional autonomy^(22)^. It is therefore reiterated that NP must be naturalized in everyday nursing.

The weaknesses experienced by the professionals participating in this research in implementing the NP were mainly related to the time it took to complete nursing records, which resulted in less time for interaction with older adults. However, as the days went by, participants progressed in completing records in less time, with the understanding that a new methodology causes challenges, which naturally tend to diminish with the experience of its daily use. Above all, the adequate and correct completion of nursing records, in order to document the NP in its execution, in fact, takes time to carry out; however, it brings professional support, in addition to subsidies for planning care, implementing care, assessing and monitoring the clinical condition, with direct improvements in continuity of care and planned treatment^(22, 23)^.

In line with this, a study identified time limitations as a complicating factor in carrying out nursing records^(23)^. However, it is crucial to raise awareness among this population about the impact of correct registration on NP consolidation. Given the work process implemented in the studied context, presenting the execution time for the development of records as a weakness is expected, given the team’s adaptation period to NP development. What should be seen with greater attention is how much this time demanded from professionals may impact quality of care. Therefore, the need to encourage nursing professionals not to give up on implementing the NP in the face of this limitation is highlighted, as it is understood that, with the frequent use of this work methodology, the time to develop it will be gradually shorter and will have a positive impact on quality of care.

### Study limitations

NP implementation assessment in a single moment (30 days) after its implementation and the regionality of the facts/collection may constitute limitations of the findings. However, through the methodological framework of action research, the challenges of implementing the NP were overcome.

### Contributions to nursing and health

The scientific evidence obtained contributes to teaching, management practice and nursing care in the gerontogeriatric context, as it demonstrates NP implementation planning, implementation and assessment in an institution caring for older adults. Furthermore, the contribution to science is highlighted, as they demonstrate the positive result of an intervention method that can serve as a model for future research and in other realities.

## FINAL CONSIDERATIONS

This research made it possible to implement NP in a faith-based senior living community for older adults, through the sequential stages of diagnosis, planning, implementation and assessment.

It was possible to implement the work method as a culture and management structure, through the organization of the local work process, construction, approval and implementation. NP materialization occurred through an instrument constructed by the researchers and tested by research participants, contributing to the knowledge, organization and continuity of care for older adults as well as to safety and professional support. Furthermore, macroprocesses were constructed as subsidies for the NP, such as the Manual of Main Care/Procedures, Nursing Care Daily Routines Manual, Abbreviations and Standardized Acronyms for Nursing Records, and SOPs.
