# Supplementary material for: Nursing Process implementation in a gerontogeriatric context: qualitative research
Source: Rev Bras Enferm. 2024 Sep 6;77(4):e20230465. doi: 10.1590/0034-7167-2023-0465 (PMC11382673; doi:10.1590/0034-7167-2023-0465)
Supplement: Translation

# Translation: Implementação do Processo de Enfermagem em contexto gerontogeriátrico: pesquisa qualitativa

**DOI:** 10.1590/0034-7167-2023-0465pt

**Authors:** Francine Casarin, Juliana Silveira Colomé, Liliane Alves Pereira, Emanuelli Mancio Ferreira da Luz, Oclaris Lopes Munhoz, Silomar Ilha

**Affiliations:** I Faculdade Integrada de Santa Maria. Santa Maria, Rio Grande do Sul, Brasil; II Universidade Franciscana. Santa Maria, Rio Grande do Sul, Brasil; III Universidade Federal do Rio Grande. Rio Grande, Rio Grande do Sul, Brasil; IV Universidade Federal de Santa Maria. Palmeira das Missões, Rio Grande do Sul, Brasil

**Keywords:** Idoso, Geriatria, Cuidados de Enfermagem, Processo de Enfermagem, Enfermagem

## Abstract

**Objetivo:**

descrever a implementação do Processo de Enfermagem em uma casa religiosa de cuidados para idosas.

**Método:**

pesquisa-ação estratégica com 19 profissionais de enfermagem e três gestores de uma casa religiosa de cuidados para idosas. A implementação ocorreu em quatro fases: diagnóstica, de planejamento, implementação e avaliação. Os dados coletados por entrevista semiestruturada e grupo focal foram submetidos à análise textual discursiva.

**Resultados:**

construíram-se as categorias centrais: Processo de Enfermagem na casa religiosa de cuidados domiciliares: diagnóstico de conhecimento e aplicação; Processo de Enfermagem na casa religiosa de cuidados domiciliares: implementação; Processo de Enfermagem na casa religiosa de cuidados domiciliares: avaliação após sua implementação.

**Conclusão:**

a implementação do Processo de Enfermagem possibilitou estruturar a gestão/organização do trabalho, contribuindo para o conhecimento, a organização e a continuidade dos cuidados para segurança e respaldo profissional.

## INTRODUÇÃO

A discussão acerca do envelhecimento populacional ocorreu mundialmente nos últimos anos, reflexo do aumento expressivo do número de pessoas idosas. Projeções indicam que, em 2050, haverá duas pessoas com mais de 65 anos para cada uma de até quatro anos no mundo^(1)^. Na realidade brasileira, esse debate é emergente, visto que, em 2025, o Brasil será o sexto maior país em concentração dessa população^(2)^. À medida que o envelhecimento ocorre, o indivíduo torna-se susceptível a vulnerabilidades sociais e/ou familiares que podem predispor a síndromes geriátricas, como incapacidade cognitiva/comunicativa, instabilidade postural, incontinência esfincteriana, imobilidade e insuficiência familiar, repercutindo na necessidade de assistência à saúde^(3)^.

Nessa perspectiva, os cuidados às pessoas idosas podem ser realizados em diferentes modalidades, como em ambientes hospitalares, em Instituições de Longa Permanência para Idosos (ILPIs) e em domicílios. O ambiente domiciliar é considerado, na maioria das vezes, o local onde a pessoa idosa passa a maior parte do seu tempo. É comum que a mesma se sinta confortável e segura ali. Assim, deve ser um espaço que propicie não somente o bem-estar, mas as condições fundamentais no cotidiano do cuidado da pessoa idosa junto aos familiares e/ou cuidadores^(4)^.

O cuidado às pessoas idosas religiosas se estrutura em casas religiosas de cuidados domiciliares, que são ambientes onde vivem irmãs que necessitam de assistência em sua senescência (processo fisiológico de envelhecimento) ou senilidade (associada a uma condição de doença). São escolhidas por proporcionarem tranquilidade, conforto e acolhimento para as irmãs idosas ou aquelas que apresentam limitações que as impossibilitem de residir em outras comunidades^(5)^.

Essas instituições devem acolher as necessidades dessa população, considerar sua trajetória de vida e conservar sua autonomia e independência, promovendo a compreensão sobre o processo de envelhecimento e as tornando protagonistas do seu cuidado^(5)^. Assim, emerge a necessidade de atuação de profissionais com vistas a um cuidado interprofissional. Entre esses, a equipe de enfermagem atua no cuidado direto e integral à pessoa idosa, sob gestão do enfermeiro, que necessita exercer suas atividades laborais em ambientes cada vez mais organizados e estruturados^(6)^.

Nesse contexto, emerge o Processo de Enfermagem (PE), um método orientador do pensamento crítico e do julgamento clínico do enfermeiro, com vistas ao direcionamento da equipe de enfermagem para o cuidado, por meio de cinco etapas: avaliação, diagnóstico, planejamento, implementação e evolução de enfermagem^(7)^. No Brasil, o PE tem sido objeto de ampla discussão na performance do enfermeiro. Embora exista o marco legal acerca de sua obrigatoriedade em todo contexto socioambiental em que ocorra o cuidado de enfermagem^(7)^, ainda há uma compreensão limitada acerca do conceito e sobre sua utilização e aplicação. Nesse sentido, é possível identificar que, em algumas realidades, o PE é implementado mais pela obrigatoriedade do que pela compressão da sua necessidade e relevância para a qualidade do cuidado de enfermagem. Tal fato dificulta a compreensão sobre as implicações práticas de sua utilização.

Para tanto, há pesquisas acerca da temática em diferentes países, como Etiópia^(8)^, Nigéria^(9)^, China^(10)^, Japão^(11)^ e Brasil^(12)^.

Porém, verifica-se escassez de pesquisas acerca dos passos para a sua implementação efetiva em cenários de cuidados domiciliares voltados às idosas religiosas. Diante disso, esta pesquisa se faz necessária por apresentar o itinerário percorrido na sua implementação, visando à qualidade do atendimento humanizado e singular, concebendo os processos biopsicossociais e espirituais da pessoa idosa. Diante do exposto, questiona-se: como implementar o Processo de Enfermagem em uma casa religiosa de cuidados domiciliares para idosas?

## OBJETIVO

Descrever a implementação do Processo de Enfermagem em uma casa religiosa de cuidados para idosas.

## MÉTODOS

### Aspectos éticos

Pesquisa autorizada pela instituição e aprovada pelo Comitê de Ética em Pesquisa da Universidade Franciscana. A participação na pesquisa ocorreu mediante assinatura do Termo de Consentimento Livre e Esclarecido (TCLE). Os participantes foram assegurados sobre o anonimato e a confidencialidade das informações e identificados pela letra P (Participante), seguida por algarismo.

### Tipo de estudo

Pesquisa-ação estratégica, na qual a transformação é previamente planejada pelo pesquisador, que acompanha os efeitos e avalia os resultados de sua aplicação^(13, 14)^. Para tanto, seguiram-se as fases de diagnóstico, planejamento, implementação e avaliação, as quais contemplam os oito passos da pesquisa-ação: identificação do problema; levantamento dos dados; análise dos dados; significado dos dados; identificação das necessidades de mudança; encontro de soluções; intervenção ação; e transformação^(14)^. No que tange à observância dos critérios de rigor metodológico para redação da pesquisa, utilizou-se o *Consolidated criteria for REporting Qualitative research* (COREQ)^(15)^.

### Cenário do estudo e período

Pesquisa realizada entre fevereiro e outubro de 2022 em uma casa religiosa de cuidados domiciliares da região central do estado do Rio Grande do Sul, Brasil, fundada em 25 de março de 1951, que oferece cuidados às religiosas do Instituto das Irmãs Franciscanas da Penitência e Caridade Cristã, que conta com duas alas, onde residem 105 idosas com diferentes níveis de dependência. O local se mantém com recursos próprios, medicações via Estado e o benefício de aposentadoria das idosas.

### População

No período investigado, atuavam no cenário 39 trabalhadores de serviços assistenciais e não assistenciais, sendo quatro enfermeiras, 17 técnicas de enfermagem, um médico assistente, um médico voluntário, uma nutricionista, uma fisioterapeuta, duas profissionais da higienização, nove copeiras e três profissionais da gestão. Foram considerados os profissionais da enfermagem e da gestão atuantes no período de coleta de dados e que preencheram os critérios de seleção.

### Critérios de seleção de participantes

Foram incluídos enfermeiros(as), técnicos(as) de enfermagem e/ou profissionais da gestão, atuantes na casa religiosa de cuidados domiciliares há no mínimo um mês, período suficiente para que já tivessem vivenciado e experienciado a realidade da instituição. Foram excluídos profissionais afastados por qualquer motivo durante a coleta dos dados. A escolha da equipe de enfermagem advém de serem os profissionais responsáveis pela operacionalização do PE^(7)^. Os profissionais da gestão foram incluídos pela compreensão de que, para o sucesso da implementação de uma nova metodologia de trabalho, torna-se necessário que a conheçam de forma integral e a reconheçam como necessária. Assim, dos 24 participantes elegíveis, 22 preencheram os critérios.

### Coleta de dados

Os dados foram coletados por meio das técnicas de entrevista semiestruturada e grupo focal (GF), em quatro fases: diagnóstico (fevereiro a abril de 2022); planejamento (agosto de 2022); implementação (setembro de 2022); e avaliação (outubro de 2022). Todas as etapas foram desenvolvidas em um local reservado na própria instituição cenário da pesquisa.

#### Diagnóstico

Na fase diagnóstica, deu-se a coleta dos dados iniciais, por meio de entrevista semiestruturada (1º momento), com os 19 profissionais de enfermagem e três da gestão. O roteiro da entrevista foi composto pela descrição dos participantes e pelas questões abertas: como é desenvolvido o processo de cuidado às pessoas idosas no local onde atua? O que você compreende por PE? Você já vivenciou/desenvolveu o PE junto a pessoas idosas residentes na casa religiosa de cuidados domiciliares em que você atua? Entrevistas foram realizadas individualmente e com agendamento prévio por dois profissionais capacitados (uma enfermeira com experiência em pesquisa qualitativa, PE e gerontologia; e uma estudante de iniciação científica que recebeu treinamento). As entrevistas tiveram duração média de 60 minutos.

A entrevista possibilitou aos pesquisadores identificar a inexistência do PE no cenário investigado, ausência de manuais, protocolos, Procedimentos Operacionais Padrão (POPs) e as fragilidades no conhecimento sobre o PE, o que conduziu à necessidade de oficinas de sensibilização sobre a temática nos aspectos conceituais e de aplicação. Foram realizadas quatro oficinas, de março a agosto de 2022, com os 22 profissionais participantes da pesquisa, conduzidas por um enfermeiro pesquisador e especialista em gerontologia. Contaram com uma apresentação expositiva dialogada e uma roda de conversa, momentos em que os participantes foram estimulados a realizar questionamentos, tirar dúvidas e manusear material vinculado ao PE^(16)^. Posteriormente, foram realizadas novas entrevistas com os participantes (2º momento), de maio a agosto de 2022, com o mesmo roteiro da entrevista anterior, para avaliar as contribuições das oficinas na construção do conhecimento dos profissionais de enfermagem e da gestão sobre o objeto de estudo.

#### Planejamento

A fase de planejamento ocorreu após a sensibilização, mediante a realização de dois GFs com 19 profissionais de enfermagem, com vistas à construção do instrumento e materiais norteadores para o desenvolvimento do PE. Consideraram-se somente os profissionais de enfermagem para esse momento, por serem os responsáveis pelo desenvolvimento do PE.

No primeiro GF, os 14 participantes receberam uma cópia da Resolução COFEN 358/2009 (válida naquele momento, substituída no ano de 2024 pela Resolução COFEN 736/2024)^(7)^ e foram convidados à leitura coletiva. Após, discutiram como sistematizar um instrumento facilitador do PE. A finalização do GF ocorreu com uma síntese das sugestões para a construção do instrumento do PE. Além desse, emergiu a necessidade da construção de manuais com informações relativas aos principais cuidados, às rotinas diárias, à padronização das abreviações para os registros de enfermagem e aos POPs. Essas sinalizações foram compiladas para construção dos materiais e do protótipo do instrumento do PE no cenário.

Para o segundo GF, contou-se com 15 participantes, para os quais a moderadora entregou uma cópia do instrumento norteador do PE. Após a aprovação pelos profissionais, o grupo discutiu a sua implementação. Ficou aprovado que cada pessoa idosa teria uma prancheta identificada, que permaneceria no posto de enfermagem, contendo prescrição médica, instrumento do PE e registro de enfermagem. Decidiram-se os horários da operacionalização das etapas do PE. Na sequência, realizou-se um treinamento com a simulação do instrumento do PE e a padronização das checagens e registros de enfermagem. Orientou-se que a utilização do protótipo do PE iniciaria no dia 05/09/22 às 00:00, com utilização pela equipe por 30 dias. Após esse período, o mesmo seria avaliado em um novo GF. Por fim, o encontro foi sintetizado a partir dos temas trabalhados, e os profissionais foram orientados quanto à implementação do protótipo construído para nortear o PE.

#### Implementação

Essa etapa ocorreu mediante a utilização, pelos profissionais de enfermagem, do instrumento norteador do PE no cotidiano de cuidados do cenário investigado. Também foi realizado um GF com os três enfermeiros para avaliação e contribuições nos materiais construídos para auxiliar o PE, organização do processo de trabalho e o estudo da NANDA-I^(16)^. No primeiro momento, o moderador apresentou uma cópia física de cada um dos materiais construídos pelos pesquisadores (com base nos materiais compilados do segundo GF), que ficaram assim denominados: Manual de Principais Cuidados/Procedimentos; Manual de Rotinas Diárias da Assistência de Enfermagem; Abreviações e Siglas Padronizadas para os Registros de Enfermagem; e POPs.

No segundo momento, realizou-se a discussão do NANDA-I^(16)^, no formato físico, para aprofundamento sobre os domínios, classes e diagnósticos. Após, os enfermeiros selecionaram os principais diagnósticos de enfermagem que representavam as condições de saúde da população do estudo. Esses foram transferidos para um quadro do *Microsoft Word*®, o qual permaneceu disponível no computador para consulta dos enfermeiros. Por fim, esse documento foi validado na síntese coletiva.

Ao término dessa fase, um dos pesquisadores, junto ao profissional responsável pela Tecnologia de Informação da instituição, informatizou o instrumento do PE e adaptou o sistema da instituição para contemplar a evolução, os diagnósticos e a prescrição de enfermagem. Também, um dos pesquisadores reuniu-se com a fiscal do Conselho Regional de Enfermagem (COREN) local para apresentar a proposta do PE, pois, no cenário estudado, o mesmo foi planejado de forma semanal, necessitando de aprovação legal. A fiscal do COREN aprovou a proposta, considerando que a premissa do PE é a adaptabilidade para atender à necessidade de cada realidade institucional.

#### Avaliação

Por fim, na fase de avaliação, ocorreu o quarto GF com os 19 profissionais de enfermagem, momento em que foram apresentados os materiais, como macroprocessos e microprocessos básicos relacionados ao PE. Foi sugerido que os participantes refletissem sobre “De que forma tais instrumentos poderão auxiliar o PE no cotidiano da casa religiosa de cuidados domiciliares?”. O registro da reflexão foi realizado em folhas de papel A4 e com canetas. Em um segundo momento, os participantes foram instigados a refletir sobre a utilização do protótipo do PE, o qual já estava em implementação há 30 dias na casa religiosa de cuidados domiciliares. Assim, no outro lado da folha distribuída anteriormente, registraram as potencialidades e fragilidades que vivenciaram na utilização do mesmo, sendo apresentado aos demais participantes.

Todos os GFs ocorreram no auditório principal da casa religiosa, local amplo e confortável, livre de influência de ruídos externos, com cadeiras dispostas em círculo, para favorecer a interação. Foram conduzidos por uma das pesquisadoras, na condição de mediadora, com a função de organizar e coordenar o GF, e uma aluna de iniciação científica, na função de observadora, a qual auxiliou no processo de gravação dos discursos, anotações e nas dinâmicas realizadas.

### Tratamento e análise dos dados

Os depoimentos pesquisados, obtidos nas entrevistas e GFs, foram audiogravados com dispositivo eletrônico de áudio MP3 e transcritos na íntegra, mecanograficamente, pelos pesquisadores, no *Microsoft Word*®. Utilizou-se a técnica da análise textual discursiva, organizada em unitarização, estabelecimento de relações e comunicação^(17)^. Inicialmente, os pesquisadores examinaram os textos com intensidade e profundidade, formando três categorias centrais, a partir do diagnóstico de conhecimento e aplicação do PE, do planejamento, implementação e avaliação. Após, cada relato foi lido e separado em diferentes unidades de sentido e, finalmente, separados em dez categorias. Por fim, apresentaram-se as compreensões atingidas a partir dos dois focos anteriores pelo processo de comunicação, resultando nos metatextos de descrição e interpretação dos fenômenos investigados^(17)^.

## RESULTADOS

Dos 22 participantes, todos eram do sexo feminino, sendo três enfermeiras, 16 técnicas de enfermagem e três profissionais da gestão, com idade entre 19 e 62 anos. O tempo de formação variou de 45 dias a 36 anos, e o tempo de experiência profissional, entre o mínimo de 30 dias e o máximo de 30 anos. Já o tempo de atuação na casa religiosa variou de 30 dias a sete anos.

Os dados produzidos nas entrevistas compuseram a fase diagnóstica e resultaram em uma categoria central e três categorias de análise. Os três primeiros GFs compuseram as fases de planejamento e implementação, resultando em uma categoria central e três categorias de análise. O quarto GF compôs a fase de avaliação do PE implementado no cenário, resultando em uma categoria central e quatro categorias de análise, conforme o Quadro 1.

**Quadro 1:** Síntese das etapas de análise dos dados. Santa Maria, Rio Grande do Sul, Brasil, 2022

Processo de Enfermagem na casa religiosa de cuidados domiciliares	
Categoria central	Categoria de análise
Diagnóstico de conhecimento e aplicação	- Presença de uma rotina, ausência do Processo de Enfermagem. - (Des)Conhecimento sobre Processo de Enfermagem antes e após as oficinas de sensibilização. - Compreensões sobre a importância do Processo de Enfermagem.
Planejamento e implementação	- Perspectivas para a implementação do Processo de Enfermagem. - Sentindo-se parte do processo. - Organização dos instrumentos necessários.
Avaliação após sua implementação	- Qualidade do cuidado: conhecimento, organização e continuidade. - Qualidade do cuidado: segurança e respaldo profissional. - Tempo na execução dos registros: fragilidade a ser superada. - Cuidado para além do biológico: necessidade de tempo para contato e convivência com as idosas.

### Presença de uma rotina, ausência do Processo de Enfermagem

Durante a fase diagnóstica da pesquisa, pôde-se perceber que os participantes identificavam a presença de uma rotina de cuidados às pessoas idosas, porém desconheciam a existência do PE.

*Existia os cuidados realizados, na grande maioria das vezes, e é nós, técnicas, que organizávamos em grupo e dividimos as tarefas. As evoluções no caderno, só escrevemos se algo da rotina das irmãs mudar.* (P1)

*Não tem nada de manual, o que a gente faz é conforme a pessoa quer. Agora que tem a enfermeira, a gente faz como ela pede para fazer. Não tem nada que norteie nosso desenvolvimento, não tem nada que diga como fazer.* (P3)

*É sempre aquela rotina. A gente segue a orientação da enfermeira e da coordenadora, que sempre está ali junto. Não tem manual, PoP, eu nunca vi, pelo menos. As orientações são realizadas nas passagens de plantão ou quando a gente precisa de alguma coisa.* (P5)

*Eu penso que tem uma rotina que a gente sempre procura seguir, sempre priorizando o bem-estar das irmãs, atender elas.* (P21)

### (Des)Conhecimento sobre o Processo de Enfermagem antes e após as oficinas de sensibilização

Ainda como diagnóstico, antes das oficinas de sensibilização, pôde-se identificar que os participantes desconheciam o conceito e a aplicabilidade do PE.

*Eu lembro que é sobre os cuidados com o paciente: cuidar do outro, se dedicar, é isso que eu lembro de quando estava estudando.* (P1)

*Olha, nunca ouvi falar, mas o nome já diz, deve ser um sistema de gestão, mais ou menos isso. Eu compreendo pela palavra, porque nunca ouvi falar. Seria um sistema de gestão, de condutas e gestão de ação, gerenciamento de tudo.* (P18)

*Não compreendo, não ouvi falar, é um sistema para toda enfermagem.* (P19)

Após a sensibilização, percebeu-se uma compreensão melhor dos participantes acerca do PE, pois as respostas foram mais coerentes com a literatura e resoluções, conforme se segue:

*O Processo de Enfermagem tem a participação do técnico. O Processo de Enfermagem, pelo que eu entendi, é organizado pelo enfermeiro: ele visita o paciente, vê o que tem que fazer para o paciente melhorar. Assim, ele organiza o cuidado em cinco etapas, que são coleta de dados, diagnóstico, planejamento, implementação e avaliação. Nós, técnicos, realizamos os cuidados prescritos pelo enfermeiro.* (P1)

*O Processo de Enfermagem é a assistência com as irmãs. Inicia com a coleta de dados, diagnóstico de enfermagem, depois faz o planejamento, traça um objetivo a partir da avaliação da paciente e, em cima disso, a gente vai realizar as condutas. A partir do que o enfermeiro avalia esse processo, vai fazer o planejamento, prescrição e, na intervenção, as técnicas vão desempenhar, para depois as enfermeiras reavaliar novamente.* (P18)

*O Processo de Enfermagem é feito pelo enfermeiro e a gente* [técnicas] *desenvolve os cuidados. O enfermeiro faz a anamnese, exame físico. O enfermeiro que prescreve os cuidados, e nós, técnicos, vamos fazer a parte do cuidado. O Processo de Enfermagem são os cuidados. A enfermeira vai lá no paciente e faz anamnese, conversa, e anotando, depois exame físico, ele observa, depois confere os diagnósticos no NANDA, vai prescrever os cuidados, e o técnico vai pra parte do desenvolvimento. Depois se faz a avaliação, se está evoluindo bem. As enfermeiras prescrevem e a gente realiza a maioria dos cuidados.* (P19)

### Compreensões sobre a importância do Processo de Enfermagem

A partir da sensibilização, percebeu-se melhor conhecimento e compreensão dos participantes acerca da importância do PE no cotidiano de trabalho junto às pessoas idosas.

*É de extrema importância. Os registros dos cuidados são tão importantes quanto os cuidados realizados, visto que ainda há algumas melhorias para se fazer.* (P2)

*Percebo a importância na casa como um instrumento de orientação na prática profissional de cada profissional da casa como um guia que está nos auxiliando a prestar um trabalho de qualidade para cada paciente/irmã da casa. É importante para podermos visualizar o que cada paciente refere e o método que será organizado o trabalho.* (P5)

*Percebo que o Processo de Enfermagem é muito importante. Os registros de enfermagem é a garantia do nosso serviço que realizamos, organização na casa e de cada uma para prestar um bom atendimento às irmãs.* (P6)

*O Processo de Enfermagem, instituído na casa, é de extrema importância, para que possa haver continuidade e legitimidade dos cuidados prestados. O processo norteia a equipe de enfermagem na execução das atividades inerentes à profissão, nos proporcionando aprimorar e desenvolver técnicas e habilidades já adquiridas, compartilhar dúvidas e sugestões, visando à saúde e ao cuidado de cada pessoa idosa.* (P11)

### Perspectivas para a implementação do Processo de Enfermagem

Nas fases de planejamento e implementação, identificaram-se as perspectivas levantadas pela equipe acerca da implementação do PE na casa religiosa. Destacou-se a compreensão de que a implementação auxiliaria na continuidade do cuidado, traria mais segurança, organização e um melhor cuidado às pessoas idosas, além de maior visibilidade, confiança e realização da equipe.

*Após a implementação do Processo de Enfermagem, vai haver a continuidade dos cuidados já prestados e uma organização, um cuidado ainda maior, uma atenção maior.* (P7)

*Vai trazer mais segurança, organização, ter método para os cuidados prestados, visibilidade nos registros de enfermagem mais organizados, específicos para cada paciente, para assistência de enfermagem mais exata e segura. A realização dos cuidados que nós, técnicas, vamos realizar, mas, conforme a prescrição do enfermeiro e da consulta de enfermagem, vai trazer mais segurança e qualidade aos cuidados, maior organização também para o ambiente.* (P9)

*Para um resultado satisfatório, precisamos de processo e ações corretas; é o que esse processo está nos fornecendo, ajudando a termos mais confiança e realização.* (P12)

*Vai qualificar o cuidado, através da implementação do processo, com a utilização dos relatórios nas rotinas. Cada trabalho realizado vai ser evoluído com data, hora, quem realizou, carimbo pela técnica responsável, nos dando respaldo ao trabalho realizado. Um trabalho em equipe com trocas de conhecimentos.* (P14)

### Sentindo-se parte do processo

Os participantes se demonstraram felizes por fazerem parte do planejamento e da implementação do PE na casa religiosa. Destacaram o aprendizado para a equipe, a melhoria na rotina diária dos cuidados e maior visualização da assistência prestada, conforme os relatos a seguir.

*Me sinto privilegiada em fazer parte desse processo, em poder ter a oportunidade de adquirir conhecimento e aprimorar cada vez mais as minhas funções, o meu aprendizado, para desenvolver um trabalho sério, com reponsabilidade e humanizado.* (P2)

*Fazer parte deste processo é um grande desafio, tanto pela valorização profissional quanto para o desenvolvimento de habilidades e cuidados, é ser parte integrante e fundamental do Processo de Enfermagem como um todo.* (P11)

*Me sinto segura por ter apoio do Processo de Enfermagem. Eu estou em adaptação, me sinto feliz em fazer parte desta experiência que, com certeza, é enriquecedora para nós da equipe de enfermagem e, para mim, como profissional desta área.* (P13)

*Aprendizado constante, talvez um comprometimento maior com as atividades do dia a dia, responsabilidade, agilidade e satisfação em aprender cada vez mais.* (P15)

### Organização dos instrumentos necessários

Durante as fases de planejamento e implementação, foram construídos e aprovados materiais para auxiliar o PE. Conforme visualiza-se a seguir, os profissionais verbalizaram que tais materiais poderão esclarecer dúvidas sobre os cuidados, padronizar e profissionalizar o trabalho, o que resultará em maior segurança e melhor cuidado às pessoas idosas.

*Os materiais construídos para auxiliar são ótimos e auxiliarão nos cuidados para tirar dúvidas no dia a dia.* (P5)

*Os materiais são fundamentais e é um diferencial, porque não tínhamos nada disso antes aqui. São muito importantes, porque podemos consultar e tirar dúvidas sobre os cuidados e sobre as siglas padrão para utilizar nos registros.* (P6)

*Acho que ter as pranchetas separadas com o nome de cada irmã, contendo o registro e o instrumento do Processo de Enfermagem, será muito bom, pois profissionalizará o nosso trabalho, melhorando ainda mais o cuidado das irmãs.* (P16)

*São muito importantes e ficaram bem ilustrativos e com uma linguagem de fácil compreensão. Os POPs, então, nem se fala, padronizaram muitos cuidados e isso traz mais segurança para nós e para as irmãs também.* (P17)

### Qualidade do cuidado: conhecimento, organização e continuidade

Após 30 dias da implementação do PE, perceberam-se contribuições de qualidade do trabalho, maior conhecimento, organização do processo laboral e ausência de fragmentação dos cuidados.

*Essa metodologia ajudou a conhecer melhor as irmãs. É mais fácil ver o que tem que fazer. Com a prescrição, tem como ver se continua com o cuidado ou se precisa outro, então é mais fácil da gente observar/visualizar e realizar os cuidados. E a relação da equipe fica mais fácil a comunicação, a gente consegue trabalhar junto muito bem. Se eu me esquecer de alguma coisa, a equipe ajuda e isso melhora a qualidade para as irmãs.* (P8)

*Cuidado clínico da enfermagem com cada irmã. Nosso trabalho como equipe mudou. Hoje, conseguimos ter uma continuidade do trabalho, a comunicação, o cuidado com as irmãs, o olhar clínico a cada uma, cada mudança, fragilidade que acontece entre elas, é isso.* (P14)

*O trabalho do enfermeiro tendo maior visibilidade e todos os cuidados aplicados e checados. Às vezes, eu paro olhando e está bem melhor a qualidade das evoluções. Elas checam e evoluem, a qualidade do trabalho das técnicas está bem melhor.* (P20)

*O cuidado mais organizado, maior visibilidade; ter as pranchetas individuais com a prescrição de enfermagem facilita a ver os cuidados a serem realizados com as irmãs. Na sequência, a gente está conseguindo visualizar o que foi realizado no turno anterior.* (P16)

### Qualidade do cuidado: segurança e respaldo profissional

O respaldo profissional e a segurança também foram potencialidades relatadas pelos participantes durante a fase de avaliação da implementação do PE, conforme se segue:

*Acho que auxiliou muito na hora da evolução mesmo e nos procedimentos. É uma segurança maior nos procedimentos realizados pela equipe anterior.* (P17)

*Um maior respaldo, porque ajuda quando a gente esquece algum cuidado. A gente olha e auxilia para saber o que fazer.* (P7)

*A prescrição de enfermagem nos traz, além do respaldo profissional, maior conhecimento, oportunidade de comunicação com o enfermeiro e também um norte quanto aos cuidados com os nossos pacientes.* (P2)

*Segurança para realizar os cuidados com as irmãs, cuidado realizado é cuidado checado. Ser respaldado ali através dos registros. E com as prescrições, a gente consegue ler e saber qual cuidado tem que fazer, qual produto usar no curativo, por exemplo. Melhor do que só um passar no plantão, a gente tem aquele respaldo.* (P22)

### Tempo na execução dos registros: fragilidade a ser superada

Uma das fragilidades a serem superadas durante efetivação do PE na casa religiosa, evidenciadas no processo de avaliação, dizem respeito ao tempo para execução dos registros de enfermagem.

*O tempo para nós é bem complicado. Os primeiros que eu fiz levei três horas. Às vezes, está fazendo exame físico e acontece alguma intercorrência com uma irmã, tem que largar o que está fazendo e ver a irmã.* (P20)

*Para mim, só tem benefícios, na verdade. Mas tem uma fragilidade, que eu notei, que é o tempo para registrar os cuidados. Um dia eu levei três horas e 20 minutos, mas agora eu estou aos poucos melhorando.* (P2)

*Falta de tempo para preencher os documentos; questão do tempo mesmo* (P7).

*Falta tempo para realização do registro de enfermagem. Às vezes, a gente se atrasa para passagem do plantão, porque uma chama, outra chama, então é o tempo.* (P5)

### Cuidado para além do biológico: necessidade de tempo para contato e convivência com as idosas

Durante o processo de avaliação do PE, os profissionais referiram que, em decorrência do tempo para realização dos registros de enfermagem, não estavam conseguindo permanecer muito tempo com as pessoas idosas, o que representava uma fragilidade.

*Falta de tempo para dar mais atenção às irmãs para realizar cuidados de qualidade.* (P6)

*A questão do tempo, porque nós, técnicas, não temos muito tempo para interagir com as irmãs, porque temos que evoluir tudo que foi feito com os cuidados.* (P10)

*A questão do tempo com as irmãs é bem corrido quando a gente está sozinho. Às vezes, as irmãs nos chamam, querem conversar, mas tem que evoluir, checar. Acho que é mais uma tensão, porque, entre um cuidado e outro, a gente conversa, mas é tudo na correria.* (P19)

*Atenção às irmãs, momento de conversar com elas, ter mais diálogo. Às vezes, é algo mais difícil, devido à demanda de tarefas diárias. Atenção com as irmãs, o momento de diálogo, de conversar, isso tem deixado um pouco a desejar.* (P22)

## DISCUSSÃO

A *interface* entre o PE e o processo de trabalho do enfermeiro se efetiva a partir da compreensão desse como um método orientador do pensamento crítico e do julgamento clínico do enfermeiro^(7)^. À medida que os enfermeiros se apropriam do PE, é possível avaliar o nível de organização, evidenciar possibilidades de melhorias e realizar o diagnóstico situacional, base do planejamento estratégico do serviço de enfermagem, possibilitando avaliar o nível de organização da assistência realizada^(12)^.

Embora já se tenham passado mais de 14 anos da publicação da resolução que apresenta a obrigatoriedade do PE, permanecem desafiadoras, em alguns cenários de atuação da enfermagem, a sua clarificação conceitual e a operacionalização prática^(12)^, evidenciadas por cenários de cuidado de enfermagem que ainda não a implementaram. Especialmente no âmbito das casas religiosas, percebe-se que a resolução^(7)^, por si só, talvez não tenha oferecido o suporte que a operacionalização do PE exige, pois muitos fatores desencadearam dificuldades práticas de implantação e implementação das etapas do PE, o que foi modificado com esta pesquisa-ação.

No contexto desta pesquisa, os profissionais de enfermagem possuíam uma rotina de trabalho empírica quanto ao modo de realizá-la, aos recursos humanos envolvidos e aos instrumentos de trabalho. Dado semelhante foi evidenciado em estudo com idosas religiosas em uma ILPI do Rio Grande do Sul^(18)^, onde o cuidado se direcionava às necessidades da pessoa idosa; entretanto, era conduzido a partir de normas e rotinas de cuidado, sem a utilização de um método específico e sólido^(18)^. Entre os fatores que contribuem para a fragmentação do cuidado nos locais que não possuem o PE instituído, podem-se citar a sobrecarga de trabalho, a dificuldade de trabalhar em equipe, a qualificação insuficiente e a ausência de materiais^(19)^. No ambiente domiciliar de cuidados, o enfermeiro possui habilidades e competências para proporcionar, junto à equipe multiprofissional, uma assistência de qualidade, tornando a pessoa idosa protagonista do seu cuidado^(20)^.

A esse respeito, durante as entrevistas junto aos participantes desta pesquisa, evidenciou-se que esses desconheciam o conceito e a aplicabilidade do PE na prática cotidiana de cuidados da pessoa idosa. Esse diagnóstico sinalizou um alerta de que o mesmo não estava sendo desenvolvido na instituição. Nesse sentido, estudo com profissionais de enfermagem atuantes com pessoas idosas identificou que os participantes reconheciam o método de orientação do cuidado a essa população. No entanto, tinham dificuldades em conceituá-lo, haja vista o déficit de conhecimentos acerca da importância do PE no cotidiano laboral, repercutindo em uma prática fragmentada baseada em rotinas^(19)^. Para tanto, são fundamentais a qualificação profissional, a educação permanente e a educação continuada da equipe para a utilização do PE. Com isso, será possível ampliar o conhecimento dos profissionais na temática para que o cuidado não se limite às práticas e demandas cotidianas^(21)^.

Identificou-se que as oficinas de sensibilização contribuíram para a compreensão dos participantes acerca do PE e da importância da sua utilização no cotidiano laboral. Investir em metodologias propulsoras de conhecimento é necessário para auxiliar na modificação de posturas e processos de trabalho. A partir do conhecimento e compreensão da sua importância, poder-se-á desenvolver com maior efetividade as etapas do PE. Nessa perspectiva, experiências de aprendizagem interativas são importantes e favorecem a prática clínica de enfermagem^(10)^.

A aplicação correta e efetiva do PE respalda cientificamente o cuidado de enfermagem e aproxima o profissional das pessoas que necessitam de assistência. Para que a atuação da enfermagem se aprimore, os envolvidos precisam ser sensibilizados e compreenderem que o gerenciamento do cuidado de enfermagem faz parte da gestão integral. Além disso, as ações devem ser planejadas e executadas em todas as fases do cuidado. Portanto, o PE é essencial para a prática no que tange à garantia da autonomia e ao fortalecimento da categoria profissional^(8)^.

Quanto à *interface* entre teoria e prática na formação acadêmica, a compreensão conceitual do PE merece destaque e deve ser estimulada por favorecer cuidados adequados e de qualidade e tratar-se de um método obrigatório que favorece um olhar singular sobre o paciente. Possuir conhecimento na temática contribui para o reconhecimento da enfermagem como profissão científica de caráter social^(8)^.

A construção de instrumentos para fortalecer a implementação do PE mostrou-se positiva e promissora no contexto avaliado, visto que contribuiu para maior respaldo e segurança aos profissionais, organização do processo de trabalho e ausência da fragmentação dos cuidados no cotidiano. Além da conscientização, obtiveram-se melhorias na percepção sobre o PE e modificações nas posturas profissionais e no processo de trabalho, o que potencializou a consolidação dos instrumentos construídos e do PE na casa religiosa de cuidados domiciliares. Logo, pode-se inferir que repercussões na qualidade do cuidado também ocorrerão, uma vez que o PE promove a melhoria do cuidado e oferece respaldo legal para a profissão por meio de seus registros, além de conferir autonomia aos profissionais, promovendo a visibilidade da profissão.

Por intermédio do PE, o protagonismo e a autonomia do enfermeiro são consolidados na aplicação de seus conhecimentos e na conquista do reconhecimento pela qualidade do cuidado prestado. Logo, profissionais de enfermagem, ao utilizarem o PE, sentem-se preparados e seguros para a realização de suas atividades, além de perceberem-se com maior autonomia profissional^(22)^. Reitera-se, dessa forma, que o PE deve ser naturalizado no cotidiano da enfermagem.

As fragilidades vivenciadas pelos profissionais participantes da presente pesquisa na implementação do PE foram relativas, majoritariamente, ao tempo para o preenchimento dos registros de enfermagem, o que resultou em menor tempo para a interação com as pessoas idosas. Contudo, com o passar dos dias, os partícipes avançaram na realização dos registros em menor tempo, com a compreensão de que uma metodologia nova ocasiona desafios, os quais naturalmente tendem a diminuir com a experiência da sua utilização diária. Sobretudo, o preenchimento adequado e correto dos registros de enfermagem, a fim de documentar o PE em sua execução, de fato, demanda tempo para a sua realização; entretanto, traz respaldo profissional, além de subsídios para o planejamento da assistência, execução dos cuidados, avaliação e acompanhamento da condição clínica, com melhorias diretas na continuidade do cuidado e no tratamento planejado^(22, 23)^.

Em consonância, estudo identificou a limitação do tempo como um dificultador para a realização dos registros de enfermagem^(23)^. No entanto, é crucial a sensibilização dessa população acerca do impacto do registro correto para a consolidação do PE. Diante do processo de trabalho implementado no contexto estudado, apresentar o tempo de execução para o desenvolvimento dos registros como uma fragilidade é algo esperado, haja vista o período de adaptação da equipe ao desenvolvimento do PE. O que deve ser visto com maior atenção é o quanto esse tempo demandado aos profissionais poderá impactar a qualidade do cuidado. Dessa forma, salienta-se a necessidade de estimular os profissionais de enfermagem a não desistirem da implementação do PE diante dessa limitação, pois entende-se que, com a utilização frequente dessa metodologia de trabalho, o tempo para desenvolvê-la será gradativamente menor e repercutirá positivamente na qualidade do cuidado.

### Limitações do estudo

A avaliação da implementação do PE em um único momento (30 dias) após a sua implementação e a regionalidade dos fatos/coleta podem se constituir limitações dos achados. Contudo, foram superados, por meio do referencial metodológico da pesquisa-ação, os desafios para implementar o PE.

### Contribuições para área da enfermagem e saúde

As evidências científicas obtidas contribuem para o ensino, a prática de gestão e a assistência de enfermagem no contexto gerontogeriátrico, pois demonstram o planejamento, a implementação e a avaliação da implementação do PE em uma instituição de cuidados a pessoas idosas. Além disso, ressalta-se a contribuição para a ciência, pois demonstram o resultado positivo de um método de intervenção que pode servir de modelo para pesquisas futuras e em outras realidades.

## CONSIDERAÇÕES FINAIS

Esta pesquisa possibilitou implementar o PE em uma casa religiosa de cuidados domiciliares para idosas, por meio das etapas sequenciais de diagnóstico, planejamento, implementação e avaliação. Foi possível implementar o método de trabalho como cultura e estrutura de gestão, por meio da organização do processo de trabalho local, construção, aprovação e implementação. A materialização do PE ocorreu por meio de instrumento construído pelos pesquisadores e testado pelos participantes da pesquisa, contribuindo para o conhecimento, organização e continuidade dos cuidados à pessoa idosa, bem como para a segurança e respaldo profissional. Ademais, foram construídos os macroprocessos como subsídios para o PE, como Manual de Principais Cuidados/Procedimentos, Manual de Rotinas Diárias da Assistência de Enfermagem, Abreviações e Siglas Padronizadas para os Registros de Enfermagem e POPs.
